# Supplementary material for: Influence of patient characteristics on chimeric antigen receptor T cell therapy in B-cell acute lymphoblastic leukemia
Source: Nat Commun. 2020 Nov 23;11:5928. doi: 10.1038/s41467-020-19774-x (PMC7683530; doi:10.1038/s41467-020-19774-x)
Supplement: Supplementary file 1 — Supplementary Information [file 41467_2020_19774_MOESM1_ESM.pdf]

Supplementary Information

Contents

Supplementary Tables

Supplementary Table 1. Tregs level between different conditioning chemotherapy groups

Supplementary Table 2. Proportion of patients with higher level Tregs between different conditioning chemotherapy groups

Supplementary Figures

Supplementary Figure 1. The function of Sino 19 cell killing CD19+ lymphoblastic cell

Supplementary Figure 2. Detection of Tregs by FCM

Supplementary Note 1

Supplementary Tables

Supplementary Table 1. Tregs level between different conditioning chemotherapy groups

	Z	*p* value*
FC vs. VDCP	-1.308	0.191
FC vs. Cy	-0.146	0.884
Cy vs. VDCP	-1.668	0.095

* Two-sided Mann Whitney U test

Supplementary Table 2. Proportion of patients with higher level Tregs between different conditioning chemotherapy groups

	Total No.	Higher Level Tregs No.(%)	*p* value*
FC	25	15(60.0%)	0.097
VDCP	5	5(100.0%)	
Cy	14	8(57.1%)	
None	2	0	
Total†	46	28(60.9%)	

* Two-sided Fisher's exact test

†Tregs data was not available for 1 patient.

Supplementary Figures

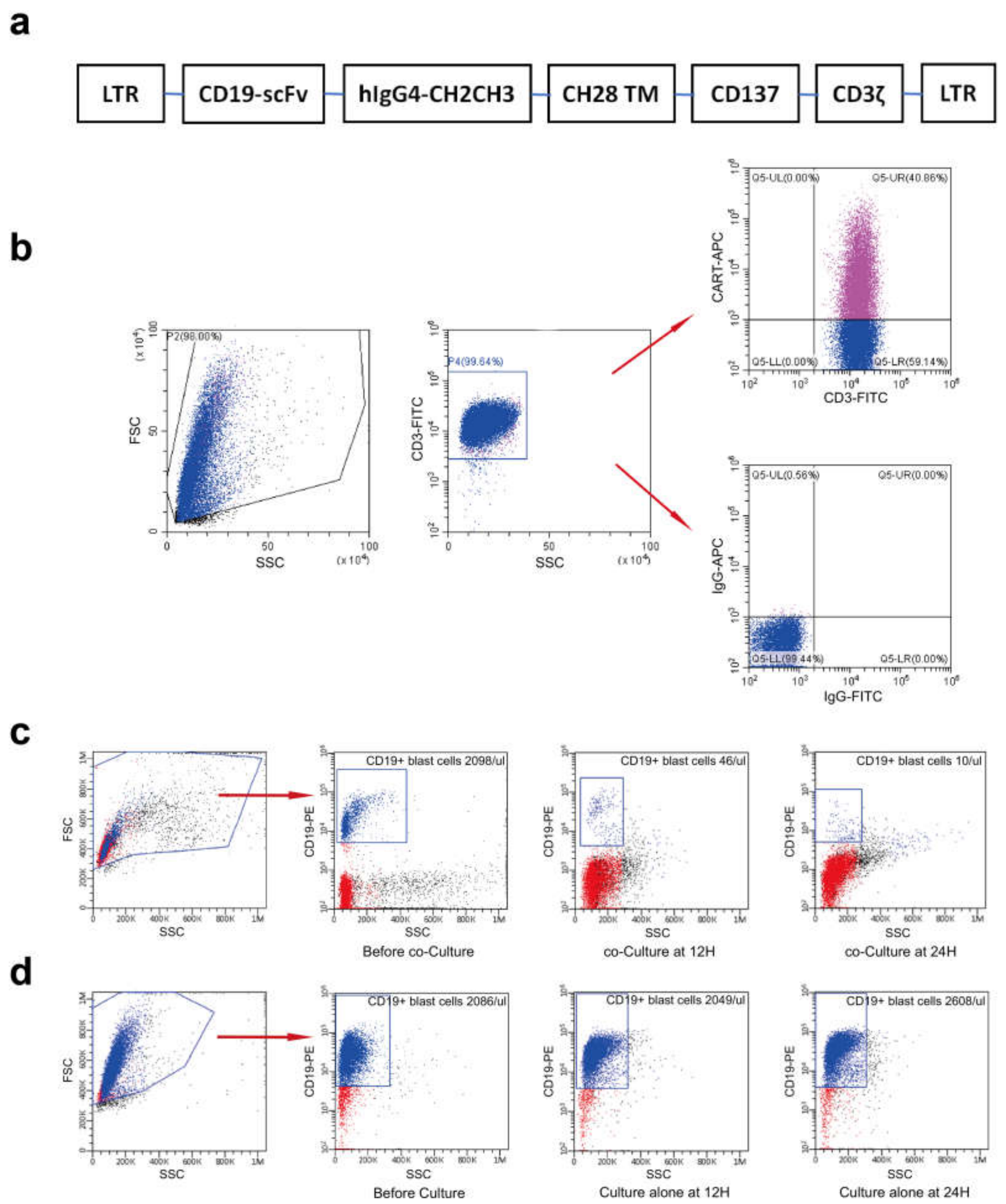

Supplementary Figure 1. The function of Sino 19 cell killing CD19^{+} lymphoblastic cell

(a) Schematics of the Sino19 cell expression constructs that were used to genetically modify T cells for this study. The LTR (retrovirus long terminal repeat), CD19-specific scFv (CD19-scFv), hIgG4-CH2CH3, CD28TM (transmembrane domain), CD137, and CD3ξ are indicated. (b) FCM assessment of CD19-CAR expression on gene modified T cells from a representative patient. (c)The CD19^{+} B lymphoblastic cells isolated from the bone marrow of a patient with r/r B-ALL were co-cultured with the autologous Sino 19 cells at a ratio of 1:1 in vitro. (d) CD19^{+} B lymphoblastic cells were cultured alone at the same consistency and conditions as a control. The flow cytometry was used to detect the number of CD19^{+} B lymphoblastic cells per-microliter before culture, at 12 and 24 hours of culture, respectively. The result suggested that at 12 or 24 hours, the amount of CD19^{+} blast cells were significantly reduced in co-cultured condition, compared to the control culture.

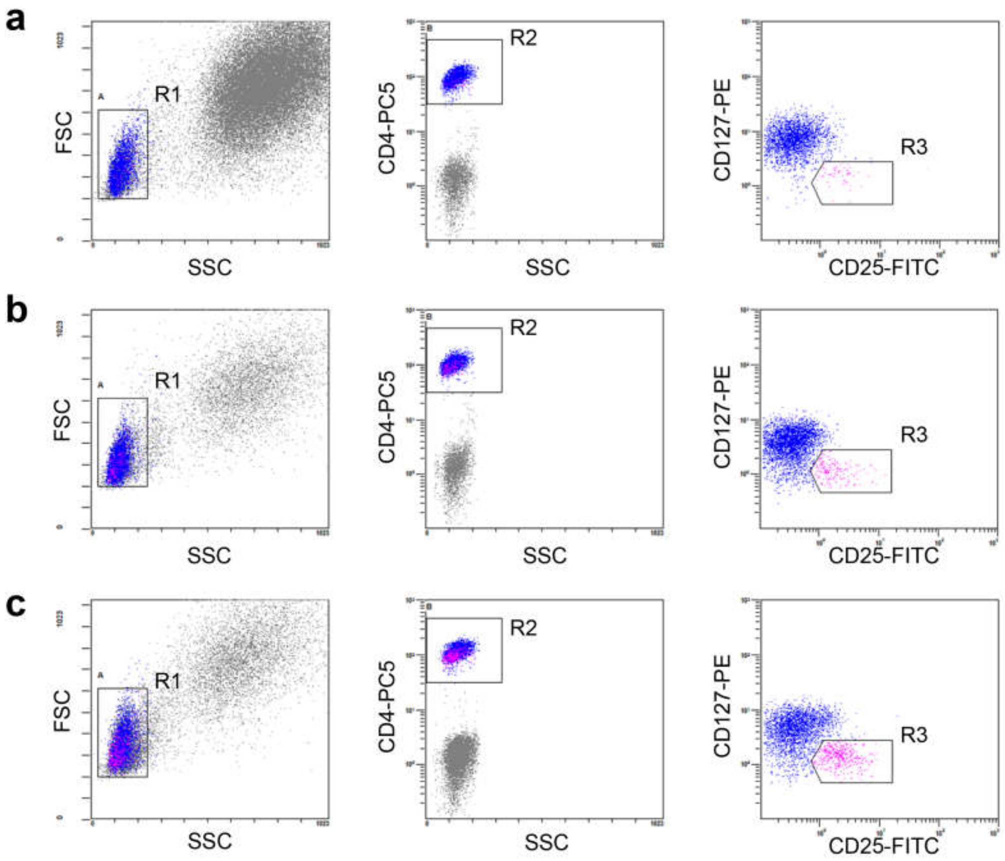

Supplementary Figure 2. Detection of Tregs by FCM

(a), (b) and (c) show the results of regulatory T cells (Tregs) for 3 different patients, the levels of blood Tregs were respectively lower, normal, and higher. From Left to right columns respectively display that forward and side scatter histograms were used to define the lymphocytes population (R1), then the expression of $CD4^+$ (R2) was assessed in the lymphocytes population, and then the expression of CD25 and CD127 was assessed in $CD4^+$ cells. Tregs were defined as $CD4^+CD25^+CD127^{low}$, and the proportion of Tregs in $CD4^+$ cells was determined (R3).

Supplementary Note 1

Study Evaluating the Efficacy and Safety with CAR-T for Recurrent or Refractory Acute Non T Lymphocyte Leukemia (EECNTL)

* This is an English translation of the main sections (including inclusion/exclusion criteria, and pre-specified outcomes) of the original study protocol; the original protocol was first approved by the Medical Ethics Committee and the Academic Committee at the Second Hospital of Anhui Medical University (SHAMU) on October 22, 2015, which is accessible at [http://www.ay2fy.com/kyb/chn_1157/content.jsp?id=8728], or from the administrator (wangjiyu1992@126.com) and the corresponding author (zzzm889@163.com) upon request.

Study Sponsor
Sinobioway Cell Therapy Co., Ltd.

Collaborator
The Second Hospital of Anhui Medical University

Study Start Date
November 2015

Brief Summary
This single-arm, multicenter Phase 2 trial will treat the patients who have recurrent or refractory acute non T lymphocyte leukemia with an infusion of the patient's own T cells that have been genetically modified to express a chimeric antigen receptor (CAR) that will bind to tumor cells that express the CD19 on the cell surface. The study will determine if these modified T cells help the body's immune system to eliminate tumor cells. The trial will also study the safety of treatment with CAR-T, how long CAR-T cells stay in the patient's body and the impact of this treatment on survival.

Detailed Description
This is a single-arm, non-randomized, multicenter Phase 2 study to evaluate the efficacy and safety of the CAR-T for recurrent or refractory acute non T lymphocyte leukemia. The study will be conducted using a phase II design. The study will have the following sequential phase: Part A (screening leukapheresis, cell product preparation, and cytoreductive chemotherapy) and Part B (treatment and follow-up). The follow-up period for each participant is approximately 35 months after the final CAR-T infusion. The total duration of the study is expected to be approximately 3 years. A total of 24 patients may be enrolled over a period of 3 years. If necessary, the sample size can be further expanded.

Study Type
Interventional (Clinical Trial)

Study Design
Intervention Model: Single Group Assignment
Masking: None (Open Label)
Primary Purpose: Treatment

Official Title

Single Arm, Phase 2, Multicenter Trial to Evaluate the Efficacy and Safety of the CAR-T cell for Recurrent or Refractory Acute Non-T Lymphocyte Leukemia

Indication

Recurrent or Refractory Acute Non-T Lymphocyte Leukemia (Refractory/relapsed B-cell acute lymphoblastic leukemia)

Intervention

Biological: CD19-targeted CAR-T cells

Study Arms

Experimental: Single arm
Name: The Chimeric Antigen Receptor T Cell Immunotherapy (CAR-T)
Dosage form：Injection
Dosage: 100ml/time; 1-5×10^6 per kg

Trial Product

CD19-targeted CAR-T cells (termed Sino19 cells)

Estimated Enrollment:

24 participants, if necessary, the sample size can be further expanded.

Estimated Study Completion Date:

May 2019

Eligibility Criteria

Inclusion Criteria:

1. All patients with acute non T lymphocytic leukemia after conventional treatment is invalid or recurrence of refractory, and by flow cytometry or pathological immunohistochemical examination, confirm the leukemia cells express can intervene molecular targets;
2. Age 3 to 75 years old, both male and female;
3. Is expected to survive more than 3 months;
4. Physical condition is good: 0-2 score ECOG score;
5. General requirements peripheral blood as basic normal (i.e., white blood cells ≥ 4.0 x10^9 L^{-1}, hemoglobin > 100 g L^{-1}, platelet count ≥ 50×10^9 L^{-1}), progress faster, in patients with special severe, fully inform the patient/guardian about the related risk to their understanding and obtain written informed consent, for such patients, peripheral blood cell index can be extended to white blood cells ≥ 2.0 x10^9 L^{-1}, hemoglobin > 60 g L^{-1}, platelet count ≥ 30 x10^9 L^{-1}. But blood T lymphocytes in peripheral blood count must be ≥ 0.2 x10^9 L^{-1};
6. No obvious abnormal heart, liver and kidney function (namely basic normal ECG; kidney function: Cr ≤ 2.0 × ULN (Upper limit of normal value); liver function: Alt/aspartate aminotransferase acuities ≤ 2.5 × ULN, Total bilirubin ≤ 2.0 × ULN), no large wounds that haven't healed on the body;
7. Into groups to participate voluntarily, good adherence can cooperate test observation, childbearing age women must be 7 days before starting treatment expert pregnancy test and the results were negative, and

signed a written informed consent form.

Exclusion Criteria:

1. Various types of T lymphocyte leukemia, etc.;
2. Organ failure, such as heart failure: Class III and IV; liver: to Child-Pugh grading of liver function grade C; kidney: kidney failure and uremia stage; lung: symptoms of severe respiratory failure; brain: a disorder of consciousness;
3. Existing serious acute infection, uncontrollable, or have fester wound and chronic infection.
4. Patients with significant graft versus host disease (GVHD) after organ transplant, or allogeneic hematopoietic stem cell transplantation;
5. Systemic autoimmune diseases or immunodeficiency disease, patients with allergic constitution.
6. Coagulation abnormalities and severe thrombosis;
7. Pregnancy and lactation women;
8. Any other chronic disease patients who have been treated with immune agents or hormone therapy;
9. Patients who are participating or have participated in other clinical trials in the past 30 days;
10. The Investigator believes the patients should not participate in this experiment.

Research Procedures：

The study was taken on the following sequential phases: screening patients, CAR-T cell manufacturing, precondition chemotherapy, CAR-T cell infusion, efficacy and adverse effects evaluation, and follow-up.

The details about screening and follow up patient are shown in the program and schedule table at the end of this protocol.

Outcome Measures

1. Primary outcome: The overall efficiency (The overall remission rate = The number of complete remission (CR) +The number of complete remission with incomplete hematologic recovery (CRi) / Total number of cases being treated)

2. Secondary outcome: Overall Survival (OS), the period between the start of treatment with CD19 CAR T cells and death from any cause; Relapse Free Survival (RFS), the duration from CR/CRi to disease recurrence; the possible influence factors affecting clinical efficacy, such as immune cell subsets, tumor load, abnormal cytogenetics and molecular biology, etc.

3. Safety: the general adverse reactions were defined and graded in 5 levels (level 0, level 1, level 2, level 3, and level 4) according to the NCI-CTCAE version 4.03 toxicity grading standard for anti-tumor drugs through systemic clinical observation, physical examination, laboratory examination and relevant special examinations.

Pre-specified Outcomes

Adverse reactions above 4 degrees are less than 10%. Overall remission rate is about 70% (at least not less than 30%). OS, RFS and possible influence factors are as results of exploration.

Investigators

Principal Investigator: Zhimin Zhai, PI; Chief physician

Contact:

Zhimin Zhai, PI; 13855147434; zzzm889@163.com
Locations: The Second Hospital of Anhui Medical University, Hefei, Anhui, China, 230601

The program and schedule table for screening and follow up patient

	Screening Period	Blood Collection	Pretreatment	Observation Period														
	-28d--21d	-20d	-20d-0d	1d	2d	3d	7d	14d	21d	28d	42d	70d	100 d	180 d	270 d	1y	2y	3y
Informed Consent Form	×																	
Peripheral Blood Collection		×																
CAR-T Infusion				×	×	×	×	×										
Vital signs	×	×	×	×	×	×	×	×	×	×	×	×	×	×	×	×	×	×
Blood Rt	×			×			×	×	×	×	×	×	×	×	×	×	×	×
Urine Rt	×			×			×	×	×	×	×	×	×	×	×	×	×	×
Bone Marrow Examination	×						×	×	×	×	×	×	×	×	×	×	×	×
Blood Biochemistry	×			×			×	×	×	×	×	×	×	×	×	×	×	×
Electrocardiogram	×							×		×	×	×	×	×	×	×	×	×
Chest Radiography	×							×		×	×	×	×	×	×	×	×	×
Leukemia Immunophenotype	×																	
Chromosome	×																	
Cytogenetic	×																	
CRP/PCT/LDH/Ig/SF	×			×			×	×	×	×	×	×	×	×	×	×	×	×
Lymphocyte Subsets	×			×			×	×	×	×	×	×	×	×	×	×	×	×
Pregnancy Test	×			×														
Contagious Disease Test	×																	
Bone Marrow MRD	×										×	×	×	×	×	×	×	×
Detection of Cytokines		×						×			×							
Adverse Event		×	×	×	×	×	×	×	×	×	×	×	×	×	×	×		
Combined Medication	×	×	×	×	×	×	×	×	×	×	×	×	×	×	×	×		
Persistence of CAR-T Cell	×		×						×	×	×	×	×	×	×	×	×	×
ECOG	×								×	×	×	×	×	×	×	×	×	×
