# Supplementary material for: Influence of patient characteristics on chimeric antigen receptor T cell therapy in B-cell acute lymphoblastic leukemia
Source: Nat Commun. 2020 Nov 23;11:5928. doi: 10.1038/s41467-020-19774-x (PMC7683530; doi:10.1038/s41467-020-19774-x)
Supplement: Supplementary file 3 — Reporting Summary [file 41467_2020_19774_MOESM3_ESM.pdf]

nature research

Corresponding author(s): Zhimin Zhai

Last updated by author(s): Oct 25, 2020

Reporting Summary

Nature Research wishes to improve the reproducibility of the work that we publish. This form provides structure for consistency and transparency in reporting. For further information on Nature Research policies, see Authors & Referees and the Editorial Policy Checklist .

Statistics

For all statistical analyses, confirm that the following items are present in the figure legend, table legend, main text, or Methods section.

n/a	Confirmed	
☐	☒	The exact sample size (*n*) for each experimental group/condition, given as a discrete number and unit of measurement
☐	☒	A statement on whether measurements were taken from distinct samples or whether the same sample was measured repeatedly
☐	☒	The statistical test(s) used AND whether they are one- or two-sided<br>*Only common tests should be described solely by name; describe more complex techniques in the Methods section.*
☐	☒	A description of all covariates tested
☐	☒	A description of any assumptions or corrections, such as tests of normality and adjustment for multiple comparisons
☐	☒	A full description of the statistical parameters including central tendency (e.g. means) or other basic estimates (e.g. regression coefficient) AND variation (e.g. standard deviation) or associated estimates of uncertainty (e.g. confidence intervals)
☐	☒	For null hypothesis testing, the test statistic (e.g. *F*, *t*, *r*) with confidence intervals, effect sizes, degrees of freedom and *P* value noted<br>*Give P values as exact values whenever suitable.*
☒	☐	For Bayesian analysis, information on the choice of priors and Markov chain Monte Carlo settings
☐	☒	For hierarchical and complex designs, identification of the appropriate level for tests and full reporting of outcomes
☐	☒	Estimates of effect sizes (e.g. Cohen's *d*, Pearson's *r*), indicating how they were calculated

Our web collection on statistics for biologists contains articles on many of the points above.

Software and code

Policy information about availability of computer code

Data collection	Clinical Data: Statistical Product and Service Solutions (SPSS) 23.0; Microsoft Excel 2010.<br>FCM Data: CXP software 2.0; MRFlow Software 1.0<br>PCR Data: LightCycler® 96 Application Software 1.1
Data analysis	Statistical analyses: Statistical Product and Service Solutions (SPSS) 23.0;<br>Power calculation: Power Analysis and Sample Size (NCSS-PASS) 11.0<br>Graph plotting: Graphpad Prism 7.0<br>FCM analyses: CXP software 2.0; MRFlow Software 1.0<br>PCR analyses: LightCycler® 96 Application Software 1.1

For manuscripts utilizing custom algorithms or software that are central to the research but not yet described in published literature, software must be made available to editors/reviewers. We strongly encourage code deposition in a community repository (e.g. GitHub). See the Nature Research guidelines for submitting code & software for further information.

Data

Policy information about availability of data

All manuscripts must include a data availability statement. This statement should provide the following information, where applicable:

- Accession codes, unique identifiers, or web links for publicly available datasets
- A list of figures that have associated raw data
- A description of any restrictions on data availability

The source data underlying Figures 1, 2, 3, 4a, 4c, 5 and Tables 1, 2, and 3, as well as the other individual de-identified participant's data that support the findings of this study are provided with this paper as a Source Data file or available at [https://doi.org/10.6084/m9.figshare.13136078.v1]. The original study protocol is accessible at [http://www.ay2fy.com/kyb/chn_1157/content.jsp?id=8728], or from the administrator (wangjiyu1992@126.com) and the corresponding author (zzzm889@163.com) upon request. An English translation of the main sections of the Study Protocol is available in the Supplementary Information file. The

remaining data is available within the Article, Supplementary Information or available from the authors upon request.
The sequence data of CAR structure is available at UniProtKB/Swiss-Prot or NCBI:
P01861.1. [https://www.uniprot.org/uniprot/P01861];
XP011510499.1.[https://www.ncbi.nlm.nih.gov/protein/XP_011510499.1];
NP001552.2. [https://www.ncbi.nlm.nih.gov/protein/NP_001552.2];
NP000725.1. [https://www.ncbi.nlm.nih.gov/protein/NP_000725.1].

Field-specific reporting

Please select the one below that is the best fit for your research. If you are not sure, read the appropriate sections before making your selection.

☒ Life sciences ☐ Behavioural & social sciences ☐ Ecological, evolutionary & environmental sciences

For a reference copy of the document with all sections, see nature.com/documents/nr-reporting-summary-flat.pdf

Life sciences study design

All studies must disclose on these points even when the disclosure is negative.

Sample size

The expected remission rate was at least 30%. According to the design of no control, single arm and two stages trail, using Simon's optimum design in 1989, under the condition of 5% error control and 80% test efficiency, the sample size of the first stage was estimated to be 10 cases, and the termination threshold of the first stage was 2 (i.e. If the number of effective patients in the first stage was less than or equal to 2, the experiment would be terminated). In the second stage, the sample size was 14 cases, and the test termination threshold was 7. If the number of effective patients was more than 7, which means that the remission rate was greater than 30%, the sample size would be further expanded. So we made a plan to enroll at least 24 patients and eventually 47 patients were treated.

Data exclusions

No data excluded.

Replication

In this study, the identification of blast cells in peripheral blood smear, or the aspirate smears from bone marrow and mass tissue, or the biopsy slides were all evaluated by three pathologists independently, the final diagnosis report was based on their consistent findings. Counting the blast cells percentage to all nucleated cells in each blood or bone marrow smear was repeated at least twice.

Randomization

This single arm clinical study was applied and randomization was not relevant.

Blinding

This single arm clinical study was applied and blinding was neither relevant nor possible.

Reporting for specific materials, systems and methods

We require information from authors about some types of materials, experimental systems and methods used in many studies. Here, indicate whether each material, system or method listed is relevant to your study. If you are not sure if a list item applies to your research, read the appropriate section before selecting a response.

Materials & experimental systems

n/a	Involved in the study	
☐	☒	Antibodies
☒	☐	Eukaryotic cell lines
☒	☐	Palaeontology
☒	☐	Animals and other organisms
☐	☒	Human research participants
☐	☒	Clinical data

Methods

n/a	Involved in the study	
☒	☐	ChIP-seq
☐	☒	Flow cytometry
☒	☐	MRI-based neuroimaging

Antibodies

Antibodies used

The following antibodies were used for flow cytometry:
FITC-CD34-mAb (Beckman Coulter, clone 581, Cat# IM1870),
PE-CD10-mAb (Beckman Coulter, clone ALB1, Cat# A07760),
FITC-CD19-mAb (Beckman Coulter, clone J3.119, Cat# A07768),
PE-CD19-mAb (Beckman Coulter, clone J3.119, Cat# A07769),
APC-CD19-mAb (Beckman Coulter, clone J3.119, Cat# IM2470),
PC5-CD45-mAb (Beckman Coulter, clone J.33, Cat# A07785),
PC7-CD45-mAb (Beckman Coulter, clone J.33, Cat# IM3548),
FITC-CD3-mAb (Beckman Coulter, clone UCHT1, Cat# IM1281U),
PC5-CD4-mAb (Beckman Coulter, clone 13B8.2, Cat# A07752),
PC5.5-CD4-mAb (Beckman Coulter, clone 13B8.2, Cat# B16491),

	FITC-CD25-mAb (Beckman Coulter, clone B1.49.9, Cat# IM0478U),<br>PE-CD127-mAb (Beckman Coulter, clone R34.34, Cat# B49220),<br>AffiniPure Goat Anti-Human IgG F(ab')2 Fragment Specific(Jackson ImmunoResearch, Cat# 109-005-006),<br>Streptavidin conjugated with APC (BD Pharmingen, Cat# 554067),<br>MACS GMP CD3 pure (Miltenyi Biotec, Cat#170-076-116),<br>MACS GMP CD28 pure (Miltenyi Biotec, Cat#170-076-117).
Validation	All antibodies are commercially available and were commercially validated.

Human research participants

Policy information about studies involving human research participants

Population characteristics	47 patients received Sino 19 cell infusion and were evaluable. The age of these 47 patients range from 3 to 72 and the median age was 22, including 23 males and 24 females; 3 (3/47, 6.4%) had primary refractory B-ALL; 9 (9/47, 19.1%) had previously received allogeneic hematopoietic stem cell transplantation (allo-HSCT) and 3 of them used blinatumomab also; 28 (28/47, 59.6%) had cytogenetic and/or molecular aberrations predicting poor prognosis; 13 (13/47, 27.7%) had active EMDs at enrollment.
Recruitment	Inclusion Criteria:<br>1. All patients with acute non T lymphocytic leukemia after conventional treatment is invalid or recurrence of refractory, and by flow cytometry or pathological immunohistochemical examination, confirm the leukemia cells express can intervene molecular targets;<br>2. Age 3 to 75 years old, both male and female;<br>3. Is expected to survive more than 3 months;<br>4. Physical condition is good: 0-2 score ECOG score;<br>5. General requirements peripheral blood as basic normal (i.e., white blood cells ≥ 4.0 x10^9/L, hemoglobin > 100g/L, platelet count ≥ 50×10^9/L), progress faster, in patients with special severe, fully inform the patient/guardian about the related risk to their understanding and obtain written informed consent, for such patients, peripheral blood cell index can be extended to white blood cells ≥ 2.0 x10^9/L, hemoglobin > 60g/L, platelet count ≥ 30 x10^9/L. But blood T lymphocytes in peripheral blood count must be ≥ 0.2 x10^9/L;<br>6. No obvious abnormal heart, liver and kidney function (namely basic normal ECG; kidney function: Cr ≤ 2.0 × ULN (Upper limit of normal value); liver function: Alt/aspartate aminotransferase acuities ≤ 2.5 × ULN, Total bilirubin ≤ 2.0 × ULN), no large wounds that haven't healed on the body;<br>7. Into groups to participate voluntarily, good adherence can cooperate test observation, childbearing age women must be 7 days before starting treatment expert pregnancy test and the results were negative, and signed a written informed consent form.<br>Exclusion Criteria:<br>1. Various types of T lymphocyte leukemia, etc.;<br>2. Organ failure, such as heart failure: Class III and IV; liver: to Child-Pugh grading of liver function grade C; kidney: kidney failure and uremia stage; lung: symptoms of severe respiratory failure; brain: a disorder of consciousness;<br>3. Existing serious acute infection, uncontrollable, or have fester wound and chronic infection.<br>4. Patients with significant graft versus host disease (GVHD) after organ transplant, or allogeneic hematopoietic stem cell transplantation;<br>5. Systemic autoimmune diseases or immunodeficiency disease, patients with allergic constitution.<br>6. Coagulation abnormalities and severe thrombosis;<br>7. Pregnancy and lactation women;<br>8. Any other chronic disease patients who have been treated with immune agents or hormone therapy;<br>9. Patients who are participating or have participated in other clinical trials in the past 30 days;<br>10. The Investigator believes the patients should not participate in this experiment.
Ethics oversight	The Medical Ethics Committee and the Academic Committee at Second Hospital of Anhui Medical University

Note that full information on the approval of the study protocol must also be provided in the manuscript.

Clinical data

Policy information about clinical studies

All manuscripts should comply with the ICMJEguidelines for publication of clinical research and a completedCONSORT checklist must be included with all submissions.

Clinical trial registration	NCT02735291
Study protocol	The original study protocol is accessible at [http://www.ay2fy.com/kyb/chn_1157/content.jsp?id=8728], or from the administrator (wangjiyu1992@126.com) and the corresponding author (zzzm889@163.com) upon request. An English translation of the main sections (including inclusion/exclusion criteria, and pre-specified outcomes) of the Study Protocol is available in the Supplementary Information file.
Data collection	From November 2015 to May 2019, the clinical data were all collected from the hematologic department at the Second Hospital of Anhui Medical University (SHAMU), which included age, gender, weight, height, the date of diagnosis, the times of chemotherapy, cytogenetic or molecular abnormalities, whether or not having allogeneic hematopoietic stem cell

	transplantation before, etc. and curative effect index including peripheral blood cell count, blast cell counts, remission time, survival time, adverse events, etc. of patients. The process all comply with the Declaration of Helsinki and the "Regulations of the People's Republic of China on the administration of human genetic resources", and under oversight of the institutional review board (The Medical Ethics Committee and the Academic Committee at Second Hospital of Anhui Medical University).
Outcomes	The primary outcome was overall remission rate which is the proportion of CR/CRi patients to all patients received CD19 CAR T cell infusion. Remission included complete remission (CR) or complete remission with incomplete hematologic recovery (CRi). The definition of CR and CRi was according to the National Comprehensive Cancer Network (NCCN) guidelines, version 1.2015. The secondary outcome was to assess the OS and RFS. Overall survival (OS) was calculated from the date of CD19 CAR T cell infusion to the date of death. The time of relapse-free survival (RFS) was from confirmation of remission to relapse. The median OS and RFS and the rate at 1 year were assessed using Kaplan-Meier approach. The safety was assessed after the infusion and at each follow-up visit. The degree of toxicities was in accordance with the National Cancer Institute Common Terminology Criteria for Adverse Events (CTCAE v4.03).

Flow Cytometry

Plots

Confirm that:

- [x] The axis labels state the marker and fluorochrome used (e.g. CD4-FITC).
- [x] The axis scales are clearly visible. Include numbers along axes only for bottom left plot of group (a 'group' is an analysis of identical markers).
- [x] All plots are contour plots with outliers or pseudocolor plots.
- [x] A numerical value for number of cells or percentage (with statistics) is provided.

Methodology

Sample preparation	The samples included bone marrow, peripheral blood, cerebrospinal fluid and CAR-T cells . The bone marrow and peripheral blood samples were stained using a whole bone marrow/blood stain-lyse method. For the other samples, cell suspensions of 1×10^6 cells/mL were prepared. All samples were anti-coagulated with heparin and examined within 4 hours. About 100ul of anti-coagulated sample was labeled with pre-conjugated monoclonal antibodies at 25∘C for 15 minutes in the dark. After incubation, red blood cells were lysed and washed twice in phosphate buffered saline (pH 7.4). Stained cells were quickly detected by flow cytometer.
Instrument	Cytomics™ FC 500 (Beckman Coulter) and Bricyte E6 (Mindray)
Software	CXP software 2.0 (Beckman Coulter) and MRFlow Software 1.0 (Mindray)
Cell population abundance	Purity was determined by evaluation of the FSC/SSC plot along with staining with the indicated antibodies. Sino 19 cells criteria for treatment included the following: cell viability ≥ 90% and CD3+ cell ≥ 90%. The CD19+ B lymphoblast cells isolated from the bone marrow were co-cultured with the autologous Sino 19 cell in vitro and the purity was more than 95% which was detected by flow cytometer.
Gating strategy	The frequency of Sino 19 cell was measured as the number of CD3+CAR+ cells out of total CD3+T cells as determined by isotype controls. Minimal residual disease was detected by flow cytometer and a target of 100,000 cells per tube was acquired. Flow cytometric analysis was performed, gating on live cells determined by CD45 and scatter characteristics. The cells were identified by CD45dim positive, and then the CD19+ B lymphoblast cells were easily distinguished by their expression of CD34, CD19 , and CD45. To analyze the relative ratio of Tregs, a lymphocyte gate was created based on the FSC versus SSC. The expression of CD4+ was assessed in lymphocytes population, and then the expression of CD25 and CD127 was assessed in CD4+ cells. Tregs were defined as CD4+CD25+CD127low, and the proportion of Tregs in CD4+ cells was determined.

- [x] Tick this box to confirm that a figure exemplifying the gating strategy is provided in the Supplementary Information.
